# Regulating Zn Ion Desolvation and Deposition Chemistry Toward Durable and Fast Rechargeable Zn Metal Batteries

**DOI:** 10.1002/advs.202205874

**Published:** 2022-12-27

**Authors:** Yuhang Zhou, Guoyu Li, Saifei Feng, Hongyu Qin, Qiancheng Wang, Fang Shen, Penggao Liu, Yanping Huang, Huibing He

**Affiliations:** ^1^ School of Chemistry and Chemical Engineering Key Laboratory of New Low‐carbon Green Chemical Technology Education Department of Guangxi Zhuang Autonomous Region Guangxi University Nanning 530004 P.R. China; ^2^ State Key Laboratory of Chemistry and Utilization of Carbon Based Energy Resources College of Chemistry Xinjiang University Xinjiang 830046 P.R. China

**Keywords:** interface engineering, ion conductor, zinc anodes, zinc ion batteries

## Abstract

The high Zn ion desolvation energy, sluggish Zn deposition kinetics, and top Zn plating pattern are the key challenges toward practical Zn anodes. Herein, these key issues are addressed by introducing zinc pyrovanadate (ZVO) as a solid zinc‐ion conductor interface to induce smooth and fast Zn deposition underneath the layer. Electrochemical studies, computational analysis, and in situ observations reveal the boosted desolvation and deposition kinetics, and uniformity by ZVO interface. In addition, the anti‐corrosion ability of Zn anodes is improved, resulting in high Zn stripping/plating reversibility. Consequently, the ZVO layer renders fast rechargeability and durable life in both Zn symmetric cells (1050 h at 10 mA cm^−2^, 1 mAh cm^−2^) and Zn/V_2_O_5_ batteries (79.1% capacity retention after 1000 cycles at 2 A g^−1^) with low electrode polarization. This work provides insights into the design of solid zinc‐ion conductor interface to enhance the interface stability and kinetics of Zn metal anodes.

## Graphical abstract

A solid electrolyte interface is rationally designed to endow fast and durable Zn anodes by simultaneously lowering the zinc ion desolvation energy barrier, improving the Zn deposition kinetics, and enabling underneath Zn plating.

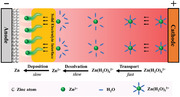

## 1 Introduction

For the past decade, lithium‐ion batteries (LIBs) have witnessed tremendous success in the consumer electronic markets, electric transportation, and grid energy storage sectors owing to their extraordinary cycling life and high energy density.^[^ ^1^ ^]^ However, the growing concerns about the safety issues and high cost encourage us to replace LIBs with safer and more reliable battery technologies.^[^ ^2^ ^]^ Rechargeable aqueous zinc‐ion batteries (ZIBs) are deemed as a rising star in recent years due to their appealing merits of low cost, non‐toxicity, intrinsic safety, and high ionic conductivity.^[^ ^3^ ^]^ Initially, the main stream to improve ZIBs performance focuses on the exploration and design of various cathode materials,^[^ ^4^ ^]^ such as manganese‐based oxide,^[^ ^5^ ^]^ vanadium‐based oxide,^[^ ^6^ ^]^ and Prussian blue analogs.^[^ ^7^ ^]^ However, the uncontrollable dendrite generation, hydrogen evolution, and corrosion reaction on the Zn anode side seriously damage the cycle stability and Coulombic efficiency of the battery, and even lead to a short circuit in the end.^[^ ^8^ ^]^

Numerous strategies have been proposed to address the aforementioned problems, including electrode design,^[^ ^9^ ^]^ separator modification,^[^ ^10^ ^]^ electrolyte modulation,^[^ ^11^ ^]^ interface engineering,^[^ ^12^ ^]^ nonaqueous electrolytes,^[^ ^13^ ^]^ alloying zinc,^[^ ^14^ ^]^ and so forth.^[^ ^15^ ^]^ Increasing electrolyte concentration to decrease H_2_O from the Zn(H_2_O)_6_^2+^ solvation shell was considered to be an effective method for reducing hydrogen evolution and inhibiting Zn dendrites growth. However, high production cost and large voltage polarization hinder the application of these salt‐concentrated electrolytes.^[^ ^16^ ^]^The construction of an artificial solid electrolyte interface (ASEI) to guide Zn deposition behavior has been proved to be another effective way to improve the reversibility of Zn anode. For instance, Liu et al. designed a zincophilic interphase of 3D‐printed g‐C_3_N_4_ to efficiently guide Zn nucleation and suppress the dendrite growth.^[^ ^17^ ^]^ Deng et al. reported a cerium‐based conversion film with compact structure for reversible and anti‐corrosion Zn metal anode.^[^ ^18^ ^]^ Yang et al. developed a 3D interconnected ZnF_2_ matrix on the surface of zinc foil as a multifunctional protective layer to inhibit hydrogen evolution reaction and dendrite growth.^[^ ^19^ ^]^ Xue et al. fabricated a 3D porous graphene‐carbon nanotubes scaffold decorated with metal–organic framework derived ZnO/C nanoparticles (3D‐ZGC) as the host for dendrite‐free Zn‐metal composite anodes, effectively reducing the Zn nucleation barriers and promoting uniform deposition.^[^ ^20^ ^]^ Although these reported artificial interface layers improve the rechargeability of Zn anodes to some extent, their high desolvation energy and sluggish Zn ion transfer kinetics would result in uneven ion distribution that triggers the “tip‐effect” and leads to the uncontrollable Zn dendrite growth, bringing out a limited electrochemical performance, especially at high Zn plating current densities.^[^ ^21^ ^]^

**Scheme 1:**
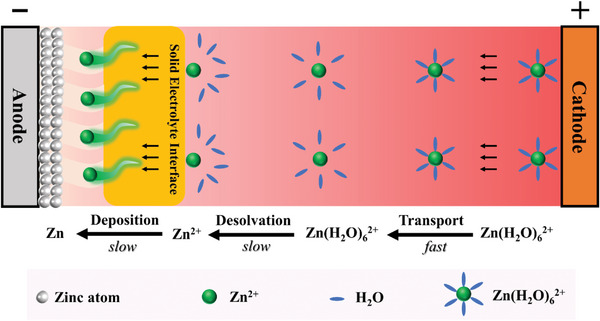
The Zn^2+^ migration behaviors before plating on Zn metal substrate with transportation, desolvation, and deposition process inside a ZIB battery.

The fast Zn deposition relies on how rapidly the Zn^2+^ can migrate between the cathode and anode. In principle, before plating on the Zn substrate, Zn^2+^ undergoes three steps during charging process (**Scheme** **1**): i) fast transportation of solvated Zn(H_2_O)_6_^2+^ in the bulk electrolyte driven by an external voltage, ii) desolvation of the solvated Zn(H_2_O)_6_^2+^ at the electrode interface, and iii) naked Zn^2+^ diffusion through SEI and subsequent deposition as metallic Zn^0^ after gaining electrons. The decisive processes (ii,iii) are usually slow due to the intrinsic high desolvation energy of solvated Zn(H_2_O)_6_^2+^ and sluggish Zn diffusion and deposition kinetics. Another issue toward the conventional interface layers is the top rather than underneath Zn metal deposits, which possess high reactivity with aqueous electrolyte, increasing potential risks of corrosion and hydrogen reaction. Therefore, rational design of a SEI that can simultaneously lower the zinc ion desolvation energy barrier and improve the Zn deposition kinetics, together with underneath Zn plating behavior, is highly desirable yet a challenge to us at present.

**Figure 1:**
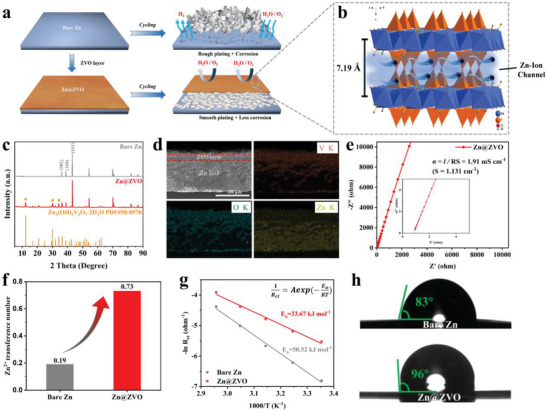
a) Schematic illustration of the Zn deposition process on bare Zn and Zn@ZVO. b) The crystal structure of Zn_3_(OH)_2_V_2_O_7_·2H_2_O. c) XRD patterns of bare Zn and Zn@ZVO. d) Cross‐sectional SEM image and corresponding EDS elemental mapping of Zn@ZVO. e) Ionic conductivity of ZVO layer. f) Zn^2+^ transference number of bare Zn and Zn@ZVO. g) Arrhenius curves of bare Zn and Zn@ZVO. h) Contact angles of 2 m ZnSO4 aqueous electrolyte on the surface of bare Zn and Zn@ZVO.

Herein, beginning with the basic principles of electrochemical Zn deposition, we have innovatively developed Zn_3_(OH)_2_V_2_O_7_·2H_2_O (ZVO) with enlarged interlayer spacing as a solid Zn‐ion‐conductor interface on Zn anode to achieve smooth and fast Zn deposition (**Figure** **1**a). The solid Zn‐ion‐conductor interface layer exhibits low activation energy of 33.67 kJ mol^−1^, high ionic conductivity of 1.91 mS cm^−1^, a large Zn^2+^ transference number of 0.73, and high electronic resistivity of 3.9 × 10^10^ Ω cm. Therefore, the ZVO layer decreases the zinc ions desolvation energy barrier, and accelerates the zinc deposition kinetics. Beside, the insulative but ion‐conductive ZVO layer enables underneath Zn plating which can efficiently avoid H_2_O/O_2_‐induced corrosion and byproducts formation. Accordingly, the symmetric cell with Zn@ZVO anode displays an excellent cycling performance over 1050 h with low voltage hysteresis at a high current density of 10 mA cm^−2^. Moreover, the Zn@ZVO/V_2_O_5_ full battery achieves a superior capacity retention of 79.1% at 2 A g^−1^ after 1000 cycles. The results indicate the promising use of solid Zn‐ion‐conductor interface to upgrade Zn anodes with enhanced kinetics, inhibited parasitic reaction, and extended cyclability.

## 2 Results and Discussion

The ZVO powder with nanosheet morphology shows characteristic X‐ray diffraction (XRD) peaks in agreement with Zn_3_(OH)_2_V_2_O_7_·2H_2_O (PDF#50‐0570), indicating the successful synthesis of the ZVO (Figure S1a,b, Supporting Information). ZVO possesses unique layered crystal structure formed by alternating connection of Zn—O octahedral layer and V—O tetrahedral layer within a large interlayer spacing of 7.19 Å between Zn—O layers (Figure 1b). This large spacing is sufficient to allow rapid Zn ion diffusion to the Zn metal anode, making ZVO a suitable carrier for Zn^2+^ transport.^[^ ^22^ ^]^ In the Fourier transform infrared spectroscopy (FTIR) spectrum of ZVO powder (Figure S2, Supporting Information), the peaks at 499.5 and 905.5 cm^−1^ are attributed to the symmetrical vibration of V—O—V and V—O—Zn, and the peak at 809.9 cm^−1^ is assigned to the asymmetric vibration of V—O—V and V—O—Zn. The peaks at 3515.5 and 1619.9 cm^−1^ can be indexed to the symmetrical vibration peak and bending vibration peak of H—O—H bond inside H_2_O, respectively, while the peak at 3187.7 cm^−1^ is regarded as the lattice vibration peak in ZVO. These results are consistent with the reported literature, and also prove that ZVO has a special layered structure composed of Zn—O layer and V—O—V layer.^[^ ^23^ ^]^ The XRD pattern (Figure 1c) shows that ZVO covers the Zn foil electrode well, with no other chemical reactions with N‐methylpyrrolidone (NMP) dispersant and polyvinylidene difluoride (PVDF) binder, which still maintain the original crystallization structure. The thickness of ZVO layer is measured to be ≈20 µm, and Zn, V, and O elements are evenly distributed into the ZVO layer (Figure 1d). Importantly, the ZVO protection layer exhibits high ionic conductivity of 1.91 mS cm^−1^ (Figure 1e), demonstrating the advantage of the artificial ZVO layer in rapid Zn^2+^ transportation based on the enlarged interlayer spacing as depicted in Figure 1b. Furthermore, the Zn^2+^ transference number is calculated as a quantitative description of the Zn^2+^ conducting ability of the ZVO layer (Figure S3a,b, Supporting Information). The Zn^2+^ transference number of the ZVO protection layer reaches up to 0.73, which is much larger than that of bare Zn with glass fiber (0.19) at room temperature (Figure 1f). The electrochemical voltage–current measurement shows that the electric resistivity of the ZVO coating layer is as high as 3.9 × 10^10^ Ω cm (Figure S4, Supporting Information), indicating its insulating nature. The high ionic conductivity coupled with electrical insulation of the ZVO interface layer is expected to enable the Zn deposition underneath the protection film. Moreover, the charge transfer resistance of the Zn|Zn symmetric cells with Zn@ZVO electrode is significantly reduced compared with bare Zn electrode at various temperatures (Figure S5a,b and Table S1, Supporting Information), demonstrating the introduced solid Zn‐ion conductor interface can accelerate ion transfer rate. In addition, the activation energy of the Zn@ZVO electrode is calculated by the Arrhenius formula as only 33.67 kJ mol^−1^ (Figure 1g), much lower than that of bare Zn (50.52 kJ mol^−1^), suggesting the decreased desolvation energy barrier and improved deposition kinetics on the Zn foil surface by the solid Zn‐ion conductor interface; thus, maybe improving the electrochemical performance of the Zn anode. The larger contact angle of Zn@ZVO anode (96°) than bare Zn (83°) implies that the ZVO layer can weaken the contact between water and Zn substrate (Figure 1h), reducing the occurrence of water‐induced parasitic reactions. In addition, the integrity of the surface coating can still be maintained after repeated folding of the electrode, showing its excellent flexibility to withstand the volume change during repeated Zn plating/stripping process (Figure S6, Supporting Information).

**Figure 2:**
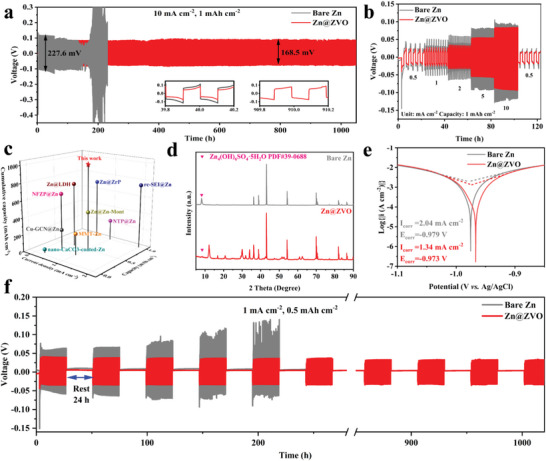
Cycling performance of Zn|Zn and Zn@ZVO|Zn@ZVO cells at a) 10 mA cm^−2^ with a capacity of 1 mAh cm^−2^. b) Rate performance of symmetric cells at current densities from 0.5 to 10 mA cm^−2^ with a capacity of 1 mAh cm^−2^. c) Comparison of cumulative plating capacity of Zn@ZVO anode with those of previously reported Zn anodes with solid electrolyte interface layers. d) XRD patterns of the bare Zn and Zn@ZVO electrode after immersion in 2 m ZnSO_4_ aqueous solution for 15 days. e) Corrosion curves of the bare Zn and Zn@ZVO. f) Electrochemical performance of Zn|Zn and Zn@ZVO|Zn@ZVO cells with alternate cycling and resting process.

The ZVO solid‐zinc‐ion conductor provides fast zinc ion transport channels with high ionic conductivity up to 1.91 mS cm^−1^ and achieves large Zn^2+^ transference number of 0.73 at the same time, which is expected to render the Zn anodes with excellent cycling performance. Galvanostatic Zn plating/stripping test is carried out to confirm the stability of Zn@ZVO electrode. At a current density of 1 mA cm^−2^ with capacity of 0.5 mAh cm^−2^ (Figure S7a,b, Supporting Information), bare Zn electrode is short‐circuited after only less than 100 h of operation, while the Zn@ZVO electrode maintains an ultra‐long cycle life of over 2000 h with a lower polarization voltage (50.1 mV) than bare Zn (117.2 mV). In addition, the nucleation overpotential (26.1 mV) is much smaller than that of bare Zn (77.2 mV), suggesting the easier Zn nucleation mode induced by ZVO interface layer. Similarly, at a current density of 2 mA cm^−2^ with capacity of 1 mAh cm^−2^, Zn@ZVO electrode also shows much more excellent cycling stability and smaller electrode polarization than the counterpart of bare Zn (Figure S8a,b, Supporting Information). Encouragingly, even at high‐rate condition (10 mA cm^−2^, 1 mAh cm^−2^), Zn@ZVO electrode can still hold stable operation of more than 1000 h without any obvious fluctuations, which outperforms that of bare Zn, indicating the superior adaptability of ZVO interface layer in manipulating uniform zinc deposition under strict circumstance (**Figure** **2**a; Figure S9, Supporting Information). In addition, the long‐term cycling performance of the Zn@ZVO electrode is competitive to those of previously reported Zn anodes with solid electrolyte interface layers. (Figure 2c; Table S2, Supporting Information). As known to us all, the lower polarization voltage ensures faster interfacial reaction kinetics between electrode and electrolyte, which would induce a lower nucleation and deposition energy barrier for Zn, promoting uniform Zn deposition.^[^ ^24^ ^]^ Figure 2b shows the rate performance comparison of symmetric cells with bare Zn and Zn@ZVO electrodes at different current densities under the same capacity of 1 mAh cm^−2^. Due to the fast zinc ion transportation, the polarization voltage of Zn@ZVO electrode at each current density keeps much smaller than bare Zn electrode. Notably, at high current densities of 5 and 10 mA cm^−2^, the bare Zn electrode shows intensive voltage fluctuation, while the Zn@ZVO electrode still remains stable during the whole stripping/plating cycles. The Zn plating/stripping kinetics is confirmed by the electrochemical impedance spectrum (EIS) of the symmetrical cells before cycling (Figures S10 and S11 and Table S3, Supporting Information). The Zn@ZVO electrode displays a much smaller semicircle than bare Zn in the high and medium frequency regions, implying a smaller charge transfer resistance at the anode interface assisted by the solid Zn‐ion conductor interface layer.

Pyrovanadate as an effective anticorrosive agent has been reported in many research works.^[^ ^25^ ^]^ Therefore, ZVO coating may play the same role in improving the chemical stability of Zn metal anode in ZnSO_4_ electrolyte. To verify its corrosion resistance, both bare Zn and Zn@ZVO electrodes are immersed in the aqueous electrolyte containing 2 m ZnSO_4_. After 15 days, the surface of bare Zn loses its metallic luster and is covered with a grayish white by‐product film (Figure S12, Supporting Information). In contrast, Zn@ZVO electrodes show no significant change, demonstrating the excellent stability of Zn anode in aqueous media by ZVO coating. The XRD patterns of the soaked electrodes (Figure 2d) also confirm that there are obvious by‐products (Zn_4_(OH)_6_SO_4_·5H_2_O, PDF#39‐0688) on the unprotected zinc surface, while there are almost none on Zn@ZVO electrodes. More importantly, the presence of the characteristic peaks for ZVO indicates the good structural stability of the introduced ZVO layer, promising the excellent cycling ability of Zn@ZVO electrodes. To further explore the corrosion resistance of ZVO coating, linear polarization experiments are performed in 2 m ZnSO_4_ electrolyte. As shown in Figure 2e, the corrosion current of Zn@ZVO decreases from 2.04 mA cm^−2^ (bare Zn) to 1.34 mA cm^−2^, and the corrosion potential of Zn@ZVO increases from −0.979 V (bare Zn) to −0.963 V. Generally, the lower corrosion current and more positive corrosion potential contribute to the better corrosion resistance.^[^ ^26^ ^]^ Similarly, the hydrogen evolution potential of the electrode is tested by linear sweep voltammetry (LSV). At the same current, Zn@ZVO shows a lower potential (Figure S13, Supporting Information), suggesting the occurrence of hydrogen evolution reaction (HER) is inhibited by ZVO coating. HER is often accompanied by the occurrence of by‐products. The more serious the HER is, the more the by‐products appear, which is consistent with that shown in Figure 2d. The reaction mechanism can be described by the following processes:^[^ ^27^ ^]^

(1) $$\;\mathrm{Zn}\to {\mathrm{Zn}}^{2+}+2e^{-}$$

(2) $$2H_{2}O+2e^{-}↔H_{2}↑+2{\mathrm{OH}}^{-}$$

(3) $$4{\mathrm{Zn}}^{2+}+{\mathrm{SO}}_{4}^{2-}+6{\mathrm{OH}}^{-}+xH_{2}O↔{\mathrm{Zn}}_{4}{\mathrm{SO}}_{4}{\left(\mathrm{OH}\right)}_{6}\cdot xH_{2}O$$

To further show its excellent anti‐corrosion ability, the charge–discharge cycle test is carried out after 7 days rest at room temperature (Figure S14a,b, Supporting Information). In the process of rest, serious corrosion and side reactions occur on the bare Zn, resulting in the generation of a large number of by‐products, which hinder the electron conduction and cause the enlarged nucleation overpotential of 111.2 mV and final short‐circuit at ≈70 h. Due to the protection of ZVO layer, the corrosion is inhibited, with much lower nucleation overpotential of 39.5 mV and longer lifespan for more than 200 h. Besides, we employ more rigorous conditions to test the long cycle stability that repeats the step of cycling for 24 h and resting for 24 h (Figure 2f). The Zn@ZVO symmetric cell can stably cycle for more than 1000 h with low polarization voltages and remain in the same state after each reboot. However, the bare Zn symmetric cell shows significantly increased polarization voltages and fluctuated stripping/plating curves after only three restarts. These results strongly prove the excellent performance of the solid Zn‐ion conductor interface layer in shielding the side reactions, which is essential to achieve enduring cycling life for aqueous Zn metal batteries.

**Figure 3:**
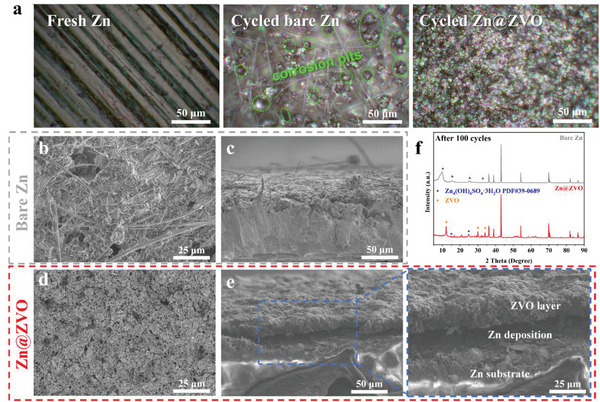
a) Optical images of fresh Zn, cycled bare Zn, and cycled Zn@ZVO. Top and cross‐section SEM images of b,c) bare Zn and d,e) Zn@ZVO (uncover part of the coating) after 100 cycles at 1.0 mA cm^−2^, 0.5 mAh cm^−2^. f) XRD patterns of the bare Zn and Zn@ZVO after 100 cycles.

High‐resolution optical images were first conducted to check the surface morphology change after cycling. As displayed in **Figure** **3**a, the fresh Zn foil showed clear grain boundaries and surface, while the cycled bare Zn electrode presented lots of corrosion pits accompanied by randomly distributed Zn deposits with metallic luster, indicating the obvious corrosion occurred during cycling. In sharp contrast, the Zn@ZVO electrode maintained a corrosion‐free surface with uniform metallic Zn deposits. In order to further explore the influence of ZVO layer on the morphology of zinc deposition, scanning electron microscope (SEM) was carried out before cycling (Figure S15a,b, Supporting Information) and after 100 cycles (Figure 3b–e) in symmetric cells. Whether from the top or cross‐sectional view (Figure 3b,c), the bare Zn surface presented a rough and loose surface filled with dendritic growth of zinc nanosheets, which may cause battery short circuits. On the contrary, Zn@ZVO remained a uniform and dense smooth surface after cycling (Figure 3d), almost as it was before cycling (Figure S15b, Supporting Information). Furthermore, its cross‐sectional SEM image (Figure 3e) demonstrates the underneath Zn deposition due to the highly ion‐conductive and insulative properties of the ZVO layer. The underneath Zn deposition can substantially alleviate corrosion reaction in aqueous media, which is necessary to endow long‐term cycling. The XRD patterns of the bare Zn and Zn@ZVO anodes after 100 cycles are shown in Figure 3f. The bare Zn electrode showed obvious by‐products (Zn_4_(OH)_6_SO_4_·3H_2_O PDF#39‐0689) with diffraction peaks at 9.9°, 15.7°, and 25.3°. This inert substance generated in the cycling would hinder the stripping and deposition of Zn electrode, resulting in irreversible loss of active Zn and seriously affecting the electrode performance.^[^ ^28^ ^]^ Notably, the ZVO solid electrolyte coating could avoid the formation of by‐products on the Zn metal effectively. In addition, element mapping reveals the presence of element S on the bare Zn (Figure S16a,b, Supporting Information) surface representing the by‐products after 100 cycles, and a much smaller amount of element S are detected on the Zn@ZVO (Figure S16c,d, Supporting Information), further confirming that the ZVO interface layer can inhibit the by‐product formation.

**Figure 4:**
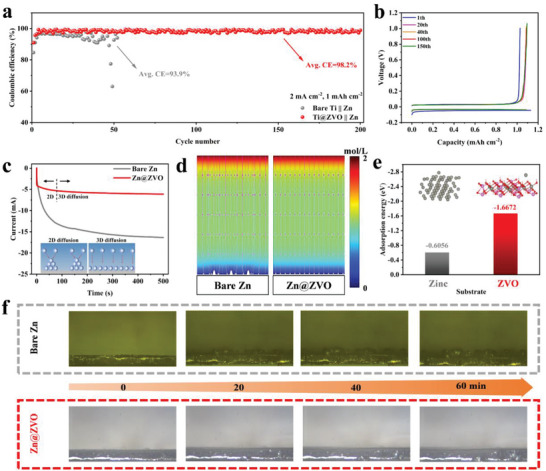
a) Coulombic efficiency and b) corresponding selected voltage profiles of the Ti|Zn and Ti@ZVO|Zn cells at 2 mA cm^−2^, 1 mAh cm^−2^. c) CA of the bare Zn and Zn@ZVO. d) COMSOL models of the Zn^2+^ concentration gradient for Zn and Zn@ZVO electrode. e) Adsorption energy of zinc ion on the bare Zn (001) and ZVO. f) In situ optical microscope observation of Zn deposition on bare Zn and Zn@ZVO electrode at 10 mA cm^−2^.

To elucidate the underlying mechanisms for the enhanced electrochemical performance by the ZVO interface layer, coulombic efficiency (CE) was measured to quantify the Zn stripping/plating reversibility by the Zn|Ti half cells configuration where Zn is dissolved from the Zn metal counter electrode and deposits onto the Ti foil during the discharge and vice versa during the charging process (**Figure** **4**a). It is evident that the bare Ti|Zn cell shows fluctuated and low average CE of 93.9% and final failure after only 40 cycles, while the Ti@ZVO|Zn cell exhibits 200 stable stripping/plating cycles with a high average CE reaching 98.2%, demonstrating the positive effect of ZVO protective layer in improving the Zn plating/stripping efficiency. Meanwhile, the nearly overlapped voltage profiles at the 20th, 40th, 100th, and 150th cycle of Ti@ZVO|Zn cell also indicate the improved Zn plating performance (Figure 4b), in contrast to its counterpart of bare Ti|Zn cell (Figure S17, Supporting Information). The improved CE can be further understood from the distinct Zn nucleation behavior and deposition pattern by the chronoamperogram (CA) test (Figure 4c). At a constant potential of −150 mV, the current of bare Zn electrode keeps increasing with time, representing the continuous 2D diffusion of Zn^2+^ on the electrode surface. Due to tip effect and the principle of lowest energy, Zn^2+^ tends to deposit on the preferential nucleation position, resulting in uneven deposition and final dendrites growth. Different from bare Zn electrodes, Zn@ZVO exhibits restricted 2D diffusion of 40 s, followed by continuous 3D diffusion with a smaller and more stable current density. The ZVO interface layer with high ionic conductivity can well adjust the diffusion mode of Zn^2+^, homogenize the ion concentration on the electrode surface, and make the Zn^2+^ diffusion rapidly change from 2D diffusion to 3D diffusion; thus, promoting the uniform and dense deposition of Zn. The molecular dynamics (MD) simulations of Zn^2+^ concentration was conducted to reveal the above different Zn deposition behavior with or without ZVO interface engineering (Figure 4d). For the bare Zn, due to the well‐known tip effect, the non‐uniform Zn^2+^ concentration distribution on the electrode surface drives more Zn^2+^ preferentially deposited on these tips during the cycling, resulting in notorious protuberances and even dendrites formation. In contrast, the ZVO solid zinc‐ion conductor can act as an artificial solid electrolyte interface on the electrode surface to homogenize the concentration of Zn^2+^ field, avoiding the tip effect; and thus, inducing uniform Zn^2+^ deposition on the electrode surface. In addition, the density functional theory (DFT) theoretical calculations results suggest that the ZVO (100) plane has a larger adsorption energy (−1.6672 eV) toward Zn than that (−0.6056 eV) of bare Zn (Figure 4e), indicating that Zn is easier to deposit on the Zn@ZVO electrode owing to the enhanced zincophilicity by the introduction of ZVO layer that reduces the nucleation barrier and gives a lower polarization voltage on the symmetric cell. Beside, the strong adsorption effect accelerates the transfer of Zn^2+^ between electrode and electrolyte, improving the Zn deposition kinetics; thus, contributing to the high rate performance. The Zn deposition behavior was further observed by an in situ optical microscope at a current density of 10 mA cm^−2^ (Figure 4f). The irregular Zn protrusions appear on the surface of the bare Zn electrode after only 20 min plating, continuously grow in the later 40 min, and may finally develop into large dendrites that would cause potential risk to the battery safety as well as performance degradation. In sharp contrast, the Zn@ZVO electrode maintains a smooth and flat surface for the entire operation process (60 min). In a word, the Zn dendrite growth is effectively inhibited by ZVO coating due to the controlled Zn deposition behavior, resulting in uniform and dense Zn plating morphology.

**Figure 5:**
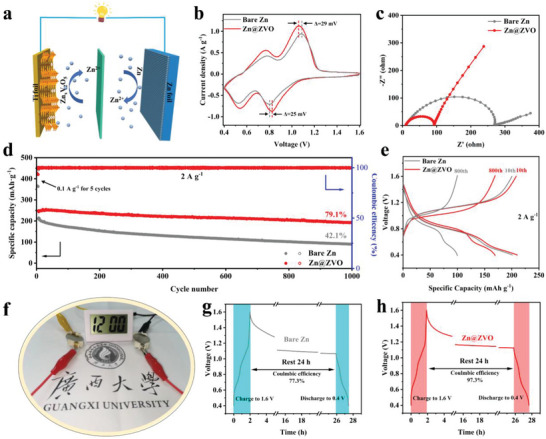
a) Zn/V_2_O_5_ battery configuration, b) CV curves, c) Nyquist plots of full cells, d) long‐term cycling at 2 A g^−1^, and e) charge–discharge profiles in the 10th and 800th cycles for bare Zn/V_2_O_5_ and Zn@ZVO/V_2_O_5_ full cell. f) A mini LCD clock run by two Zn@ZVO/V_2_O_5_ full cells in series. Self‐discharge performance of full cells with g) bare Zn and h) Zn@ZVO electrode.

Encouraged by the excellent rechargeability of Zn@ZVO anode, we finally construct a Zn/V_2_O_5_ full cell (**Figure** **5**a) to explore the effectiveness of our proposed strategy in practical application. V_2_O_5_ nanobelts are successfully synthesized by a simple solution treatment method (Figure S18, Supporting Information). The cyclic voltammetry (CV) curves of Zn/V_2_O_5_ and Zn@ZVO/V_2_O_5_ full cell show two typical redox peaks that represent the Zn^2+^ intercalation/deintercalation reaction of V_2_O_5_, indicating the ZVO layer has negligible effect in the redox process (Figure 5b). However, the Zn@ZVO/V_2_O_5_ full cell displays smaller voltage polarization compared to Zn/V_2_O_5_ full cell, suggesting its good reversibility by the ZVO interface. Consequently, higher capacities can be achieved at 0.1, 0.5, 1, 2, and 5 A g^−1^ in Zn@ZVO/V_2_O_5_ full cell than Zn/V_2_O_5_, demonstrating its better rate capability (Figure S19, Supporting Information). The ion/electron transfer kinetics of full cells are analyzed by EIS (Figure 5c). Obviously, the semicircle of Zn@ZVO/V_2_O_5_ full cell in the middle and high frequency region is much smaller than that of Zn/V_2_O_5_ full cell, which represents lower charge transfer resistance. The ZVO coating with high ionic conductivity promotes the diffusion process of Zn^2+^; thus, facilitating the Zn deposition/exfoliation processes. Therefore, the artificial solid electrolyte interface coating with high ionic conductivity is expected to improve the long‐term cycle performance of the full cell (Figure 5d). After over 1000 cycles, Zn@ZVO/V_2_O_5_ full cell maintains a high discharge capacity of 192.1 mAh g^−1^ with a capacity retention of 79.1%. The excellent performance is comparable to that of the previous literature (Table S4, Supporting Information). However, the bare Zn/V_2_O_5_ full cell displays a rapid decline in the discharge capacity, with only 90.2 mAh g^−1^ after 1000 cycles (42.1% capacity retained), which may be related to the serious side reactions and uncontrollable dendrite growth on the bare Zn anode. The corresponding charge–discharge profiles in the 10th and 800th cycles of both full cells are shown in Figure 5e. Obviously, the Zn@ZVO/V_2_O_5_ full cell exhibits a lower voltage polarization, which is consistent with cyclic voltammetry of the full cells. Such remarkable cycling performance enables two Zn@ZVO/V_2_O_5_ full coin cells in series to run a mini liquid crystal display (LCD) clock (Figure. 5f). The battery shelf life is also a key index for the evaluation of aqueous battery system. Therefore, the self‐discharge behavior is monitored to observe the alleviated side reactions in the battery storage time. After 24 h rest, a CE of 97.3% is achieved in Zn@ZVO/V_2_O_5_ cells (Figure 5g), while only 77.3% capacity is retained for the cells with bare Zn (Figure 5h).

## 3 Conclusion

In conclusion, ZVO as a solid zinc‐ion‐conductor interface with low desolvation energy, high deposition kinetics, and underneath Zn plating was designed to improve the rechargeability of aqueous Zn metal anode in this work. Benefiting from the rapid diffusion pathway of Zn^2+^ by the enlarged layer spacing in ZVO molecules, ZVO layer enables fast and uniform Zn deposition. Beside, the ZVO coating can protect Zn metal in aqueous electrolyte media for alleviated corrosion. Therefore, a dendrite‐free and corrosion‐free Zn metal anode can be achieved. As a result, the Zn@ZVO symmetric cell shows an excellent cycling stability for over 2000 h with a low polarization voltage at 1 mA cm^−2^, 0.5 mAh cm^−2^. Meanwhile, the reversibility of Zn anode can be greatly improved by the ZVO coating with average Coulombic efficiency reaching 98.2% for over 200 cycles. Furthermore, the Zn@ZVO/V_2_O_5_ full cells deliver competitive cycling performance with 79.1% capacity retention after 1000 cycles at 2 A g^−1^. The adoption of solid zinc‐ion‐conductor interface may provide a fresh perspective for achieving high‐performance aqueous Zn metal batteries.

## Conflict of Interest

The authors declare no conflict of interest.

## Supporting information

Supporting Information

## Data Availability

Research data are not shared.
