# Supplementary material for: Regulating Zn Ion Desolvation and Deposition Chemistry Toward Durable and Fast Rechargeable Zn Metal Batteries
Source: Adv Sci (Weinh). 2022 Dec 27;10(6):2205874. doi: 10.1002/advs.202205874 (PMC9951317; doi:10.1002/advs.202205874)
Supplement: Supplementary file 1 — Supporting Information [file ADVS-10-2205874-s001.pdf]

WILEY-VCH

Supporting Information

Regulating Zn Ion Desolvation and Deposition Chemistry towards Durable ad Fast Rechargeable Zn Metal Batteries

*Yuhang Zhou, Guoyu Li, Saifei Feng, Hongyu Qin, Qiancheng Wang, Fang Shen, Penggao Liu, Yanping Huang *, Huibing He**

Table of Contents

Experimental section 3
Preparation of ZVO Nanosheets 3
Preparation of Zn@ZVO 3
Preparation of V_2O_5 nanobelts 3
Materials characterizations 3
Electrochemical measurements 3
Computational detail 4
Supporting Figures and Tables 6
Figure S1 6
Figure S2 7
Figure S3 8
Figure S4 9
Figure S5 10
Figure S6 11
Figure S7 12
Figure S8 13
Figure S9 14
Figure S10 15
Figure S11 16
Figure S12 17
Figure S13 18
Figure S14 19
Figure S15 20
Figure S16 21
Figure S17 22
Figure S18 23
Figure S19 24
Tbale S1. 25
Table S2 26
Table S3 27
Table S4 28
Reference 29

Experimental Section

Preparation of ZVO Nanosheets

ZVO Nanosheets were synthesized by a simple solution precipitation method. First, 0.15 mol $ZnSO_4 \cdot 7H_2O$ and 0.1 mol $NaVO_3$ were dissolved into 50 ml deionized water and stirred until clear. The above two solutions were mixed for 12 h by magnetic stirring. The pale yellow products were available by centrifugation separation and by washing several times with deionized water. Then the products were vacuum dried at 60 °C for 12h, the ZVO nanosheets were obtained.

Preparation of Zn@ZVO

The Zn foil was washed by ultrasound with ethanol and deionized water respectively before use. ZVO and PVDF were mixed with a weight ratio of 8:2 in NMP dispersant. The slurry was coated onto the the Zn foil by the doctor blading method and dried at 70 °C for 24 h under vacuum.

Preparation of V_2O_5 nanobelts

V_2O_5 nanobelts were prepared according to the previous work.[1] 1 g commercial V_2O_5 power was added into 100 ml of 2M NaCl solution with intense magnetic stir for 72 h at room temperature. Then, the precipitate was washed several times with deionized water and alcohol. Finally, the brown precipitate was dried at 80 °C for 24 h under vacuum and the V_2O_5 nanobelts were obtained.

Materials characterizations

Powder X-ray diffraction (XRD) patterns were carried out on a D/Max-III X-ray diffractometer (Rigaku Co., Japan) with Cu Kα radiation (λ= 1.5406 Å). The morphology of the materials was collected on a field emission scanning electron microscope (FE-SEM, SU8220 Hitachi Corp, Japan) equipped with an energy dispersive X-ray spectrometer (EDS). The *in-situ* optical images were collected on a DMM-900C metallographic microscope (Caikon optical instrument Co., Shanghai) by using an optical electrochemical cell (Hefei in-situ Technology Co., Ltd).

Electrochemical measurements

CR2025-type coin Zn-Zn symmetric cells were assembled with two identical electrodes of bare Zn foil, Zn@ZVO(Φ = 12 mm), 2 M $ZnSO_4$ electrolyte and glass fiber separators (Φ = 19 mm). V_2O_5 nanobelts were mixed with Super P and PVDF with a ratio of 7:2:1 in NMP dispersant. The slurry was coat onto Ti foil (30 μm, 99.99%) by the doctor blading method and dried at 70 °C for 24 h under vacuum. The electrode was cut into round disks (Φ = 10 mm) as the cathode for the tests of full cells (The mass loading of V_2O_5 active material in

electrodes was approximately 1.5 mg cm^{-2}). The cathode and anode electrodes were separated by glass fiber separators (Φ = 19 mm). 2 M $ZnSO_4$ aqueous solution was used as the electrolyte for all the coin cells in this work, which were assembled in the air atmosphere. Galvanostatic measurements were carried out on the NEWARE battery tester (MIHW-200-160CH, Shenzhen) at room temperature. The electrochemical characterizations were all tested on a electrochemical workstation (Interface 1010E, Gamry, USA).

The Ionic conductivity (σ) was tested via EIS measurement (100 kHz to 0.01 Hz). ZVO interlayer was sandwiched between two stainless steel spacers to measure EIS. Ionic conductivities were calculated according to the following equation:

$$\sigma = \frac{l}{RS}$$

where R represents the resistance according to EIS measurement, *l* represents the thickness (20 μm), and S is the Zn@ZVO electrolyte area (1.131 cm^2).

Computational detail

The interlayer spacing of between Zn-O layers was caculated by the Bragg equation (formula (1)):

$$d = \frac{\lambda}{2\sin\theta} \quad (1)$$

where d represents crystal plane, λ represents the wavelength of the X-ray beam (0.154nm), θ represents the diffraction angle.

DFT calculations were conducted through the Vienna ab initio Simulation Package (VASP) with the projector augment wave method. Generalized gradient approximation of the Perdew-Burke-Ernzerhof (PBE) functional was used as the exchange-correlation functional. The cutoff energy was set as 500 eV, and structure relaxation was performed until the convergence criteria of energy and force reached 1×10^{-4} eV and 0.02 eV $Å^{-1}$, respectively. A vacuum layer of 15 Å was constructed to eliminate interactions between periodic structures of surface models. The van der Waals interaction was amended by the zero damping DFT-D3 method of Grimme.

The adsorption energy (ΔE_{ads}) of Zn adsorption on surface is defined as :

$$\Delta E_{ads} = E(*Zn) - E(*) - E(Zn)$$

where E(*Zn) and E(*) are the total energy of surface systems with and without Zn atom, respectively, E(Zn) is the energy of an isolated Zn atom. According to this definition, negative adsorption energy suggests that the adsorption process is exothermic and the adsorption system is thermodynamically stable. Contrarily, a positive value corresponds to an endothermic and unstable adsorption.[2]

Two physical models of Electrostatic and Transport of Diluted Species were coupled for FEM based on the following partial differential equations:

$$E = -\nabla V$$

$$J_i = -\mathrm{D}_i \nabla c_i + \mathrm{z}_i \mu_{mj} F c_i E$$

$$\frac{\partial c_i}{\partial t} = -\nabla J_j$$

where E is the electric field (V), V is the electric potential (V), J_i is the flux vector of component i (mol m^{-2} s^{-1}), D_i is the diffusion coefficient of component i (m^2 s^{-1}), c_i is the concentration of component i (mol m^{-3}), z_i is the charge number of component i, $\mu_{m,j}$ is the ion mobility of component i (mol s kg^{-1}) and F is the Faraday constant.

Supporting Figures and Tables

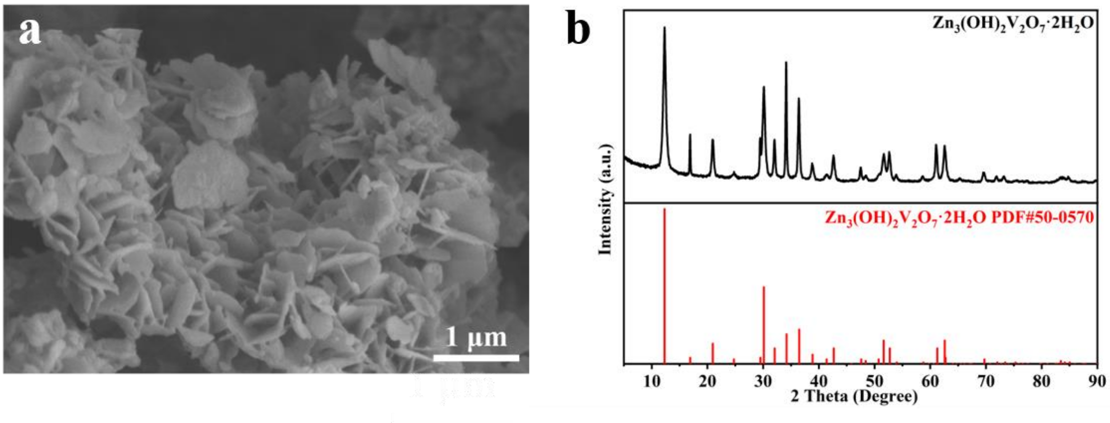

Figure S1. (a) The SEM image and (b) the XRD pattern of ZVO powder.

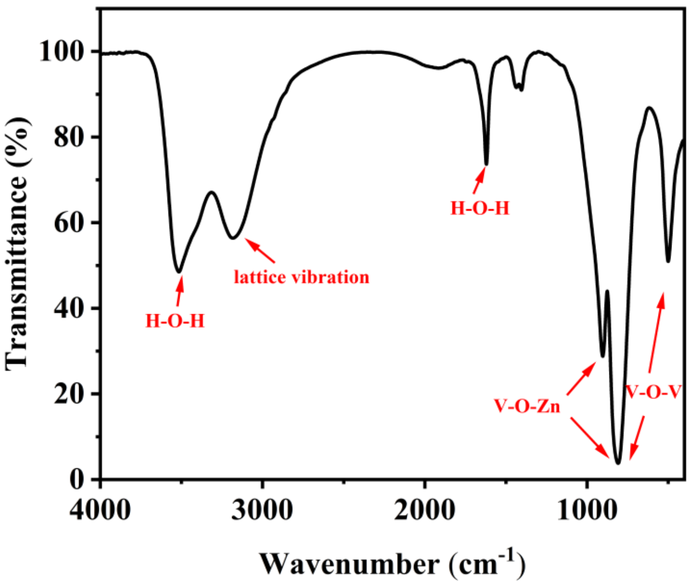

Figure S2. The FTIR pattern of ZVO.

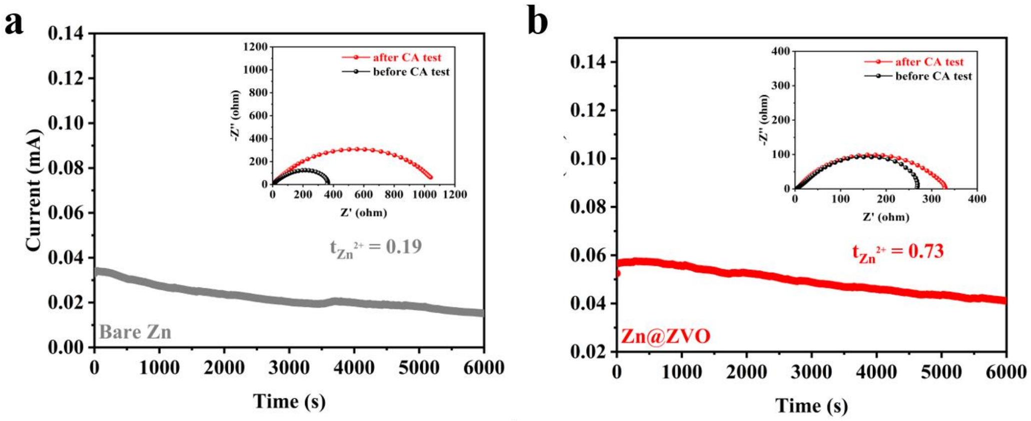

Figure S3. Linear polarization curves and the impedance spectra before and after the measurement. (a) Bare Zn. (b) Zn@ZVO. The transference number of Zn^{2+} ($t_{Zn^{2+}}$) was evaluated by EIS of symmetrical cells before and after chronoamperometry (CA) tests, as indicated by the following equation:

$$t_{Zn^{2+}} = \frac{I_S(\Delta V - I_0 R_0)}{I_0(\Delta V - I_S R_S)}$$

where ΔV is the applied voltage polarization (10 mV), I_0 and R_0 are the initial current and the resistance, and I_S and R_S are the steady current and the resistance.

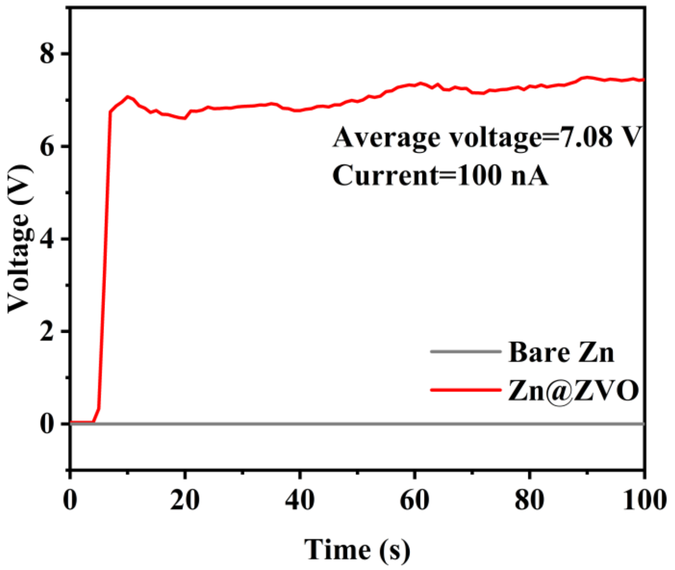

Figure S4. Conductivity measurements of bare Zn and Zn@ZVO using blocking electrodes with an applied current of 100 nA. According to the following formula of $\rho = \frac{\mathrm{RS}}{l} = \frac{US}{Il}$, the resistivity of Zn@ZVO electrode is estimated as 3.9×10^{10} Ω cm.

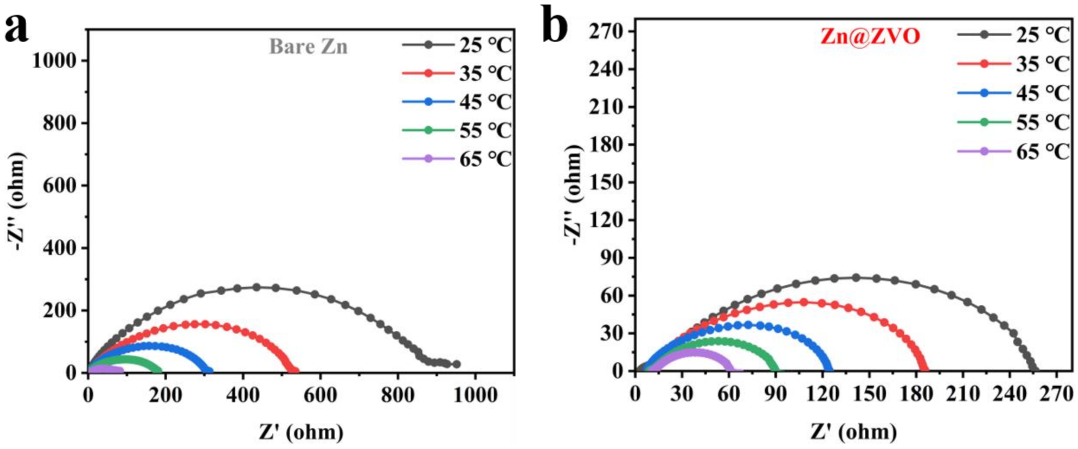

Figure S5. The EIS of symmetric cells with (a) bare Zn and (b) Zn@ZVO at various temperatures.

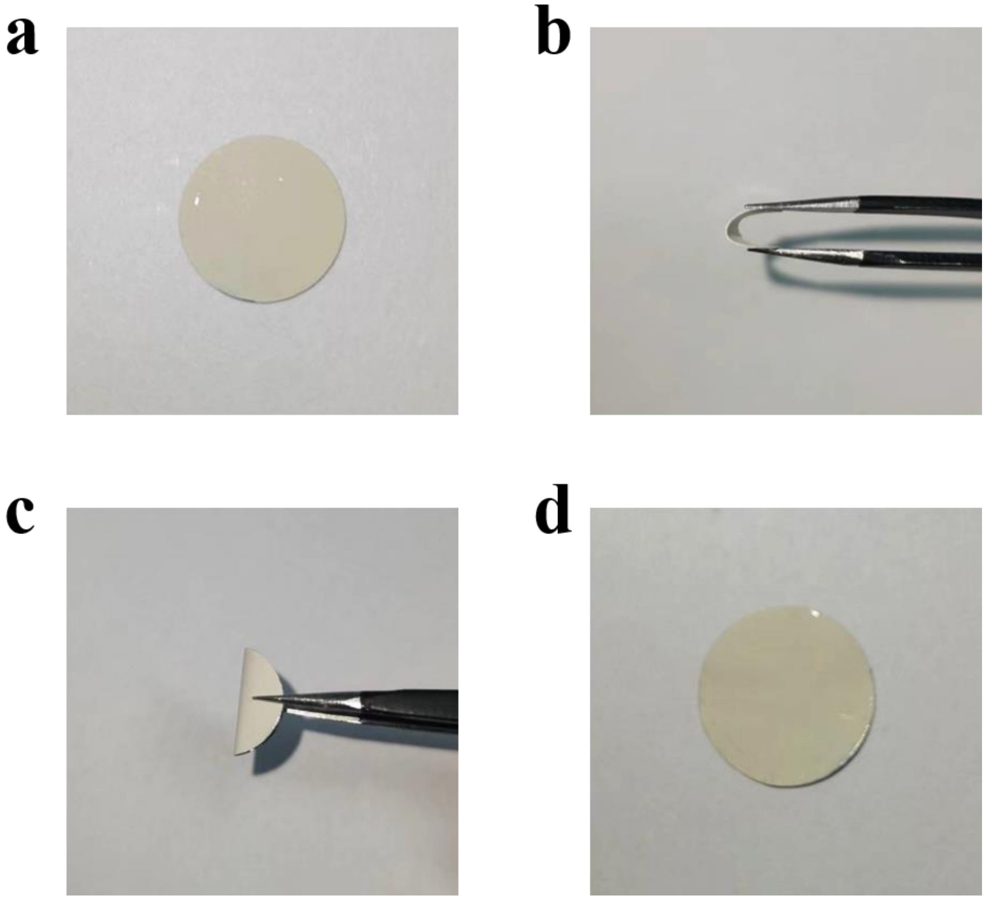

Figure S6. Optical images of Zn@ZVO (a) before, (b,c) after bending and (d) repeated bending.

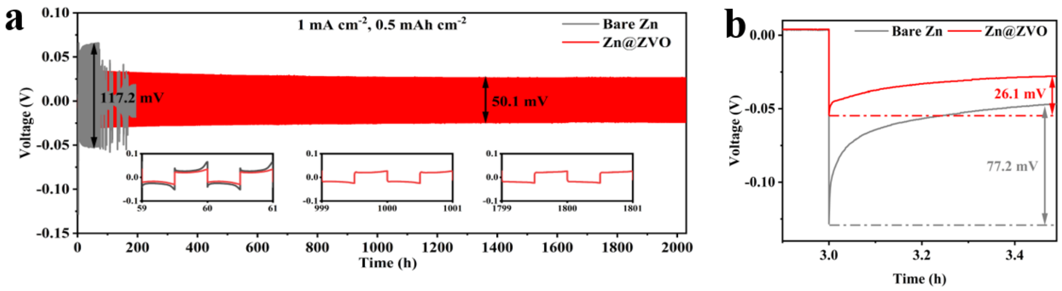

Figure S7. (a) Cycling performance of Zn|Zn and Zn@ZVO|Zn@ZVO cells at 1 mA cm^{-2}, 0.5 mAh cm^{-2} (b) Voltage profiles at the 1st plating of (a)

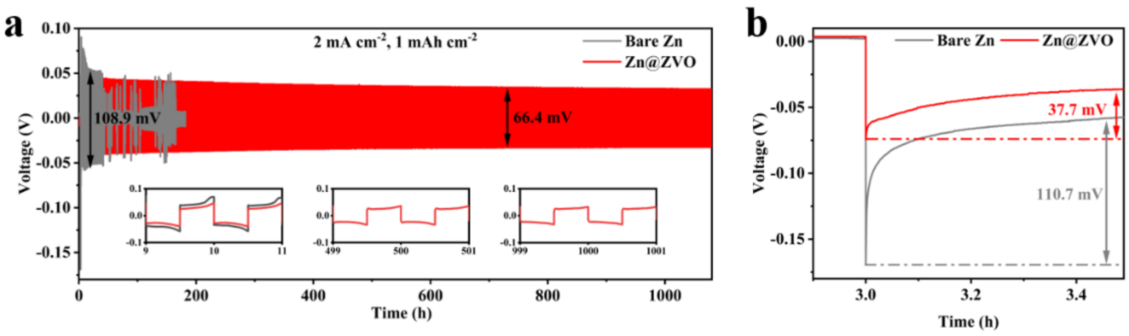

Figure S8. (a) Cycling performance of Zn|Zn and Zn@ZVO|Zn@ZVO cells at 2 mA cm^{-2}, 1 mAh cm^{-2} (b) Voltage profiles at the 1st plating of (a)

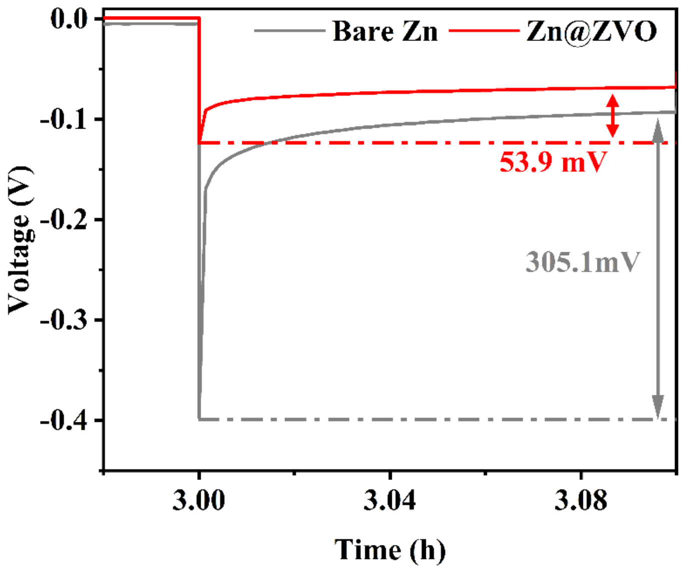

Figure S9. Voltage profiles at the 1st plating of Figure 2a.

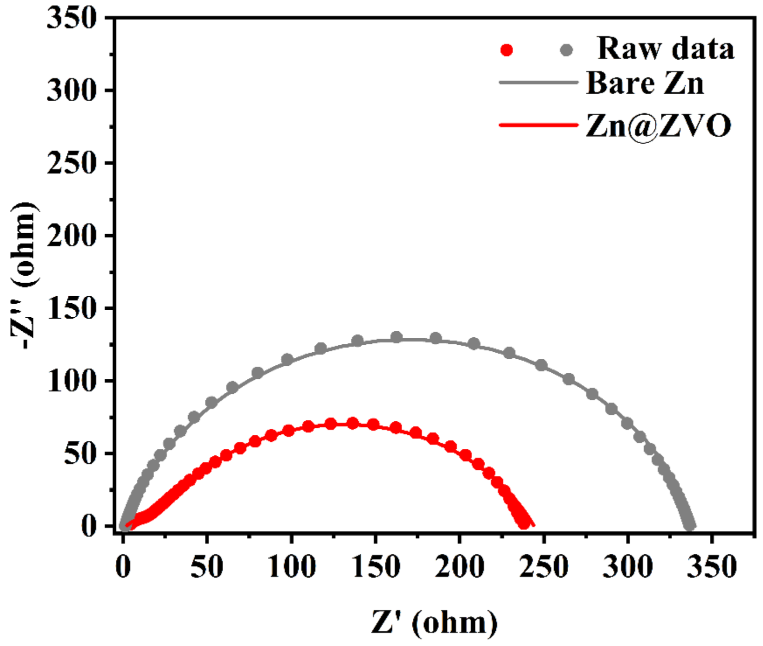

Figure S10. Nyquist plots of Zn|Zn and Zn@ZVO|Zn@ZVO symmetric cells.

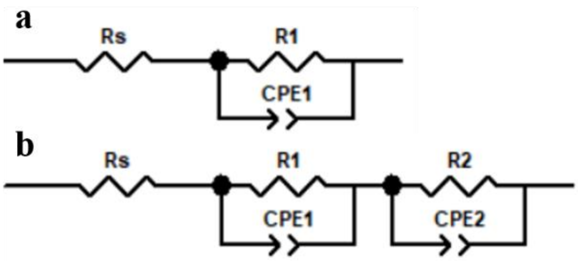

Figure S11. Fitted EIS model of (a) bare Zn|Zn and (b) Zn@ZVO|Zn@ZVO symmetric cells.

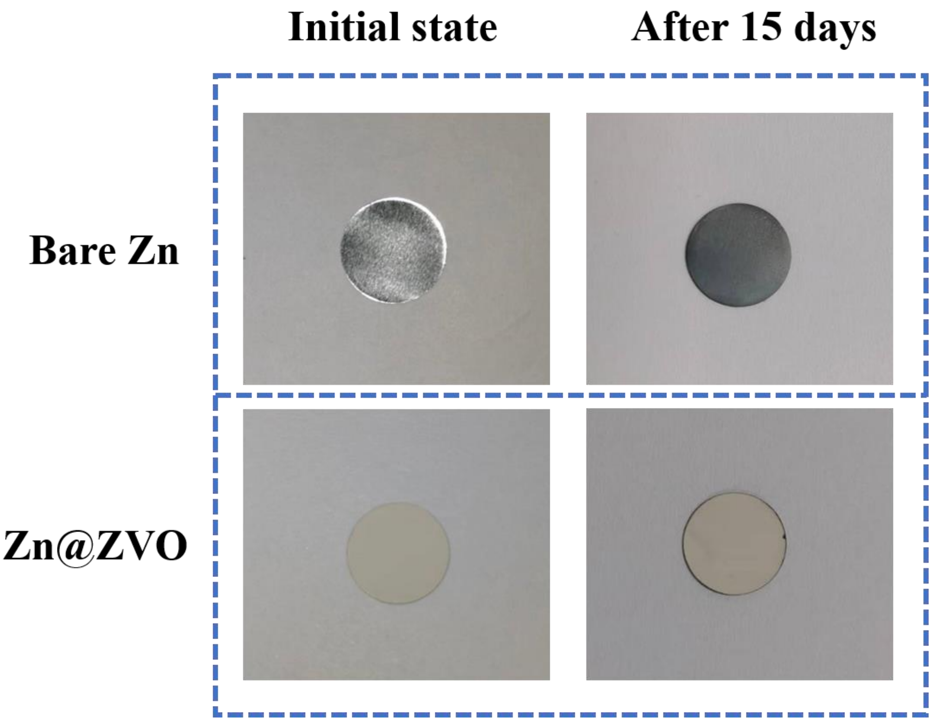

Figure S12. Photographs of corrosion morphology for the bare Zn and Zn@ZVO electrodes after immersion in 2 M $ZnSO_4$ aqueous solution for 15 days.

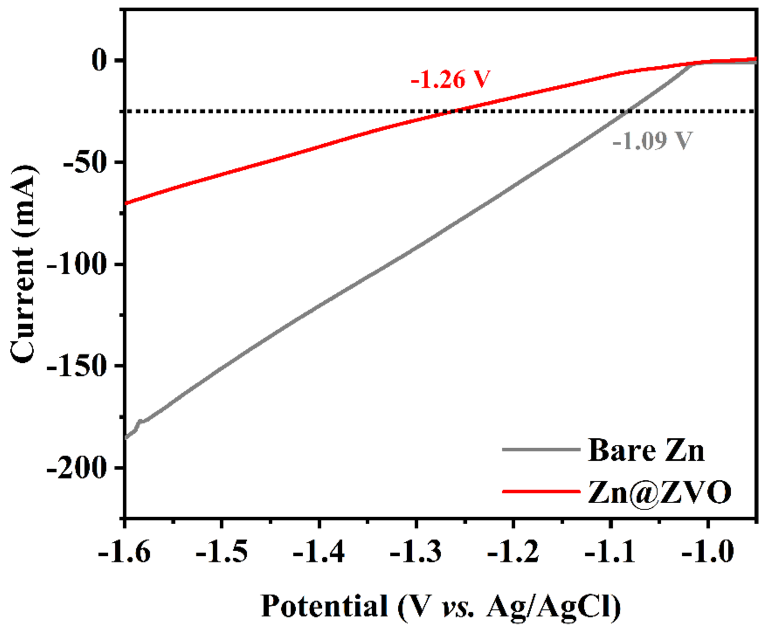

Figure S13. Linear sweep voltammetry curves of the bare Zn and Zn@ZVO.

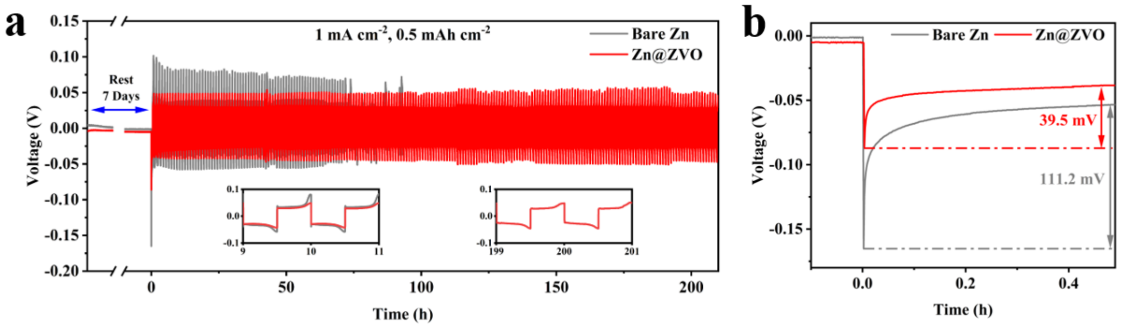

Figure S14. (a) Cycling performance of Zn|Zn and Zn@ZVO|Zn@ZVO cells after 7 days rest. (b) Voltage profiles at the 1st plating of (a).

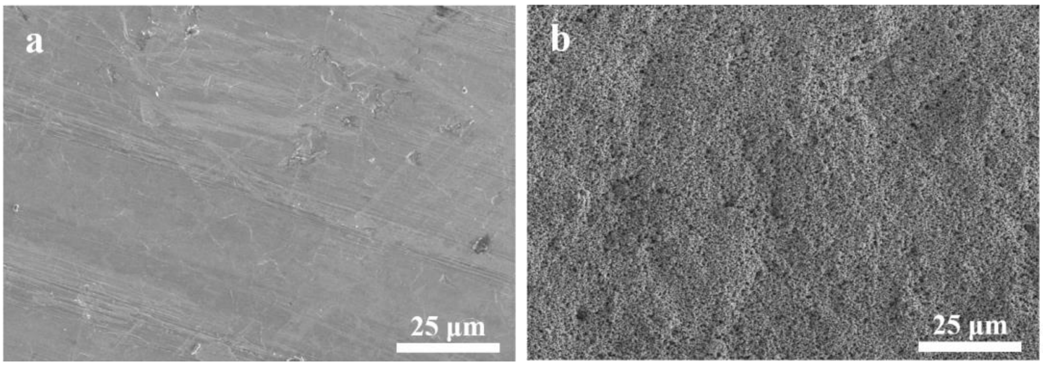

Figure S15. The top view SEM image of (a) bare Zn and (b) Zn@ZVO before cycling.

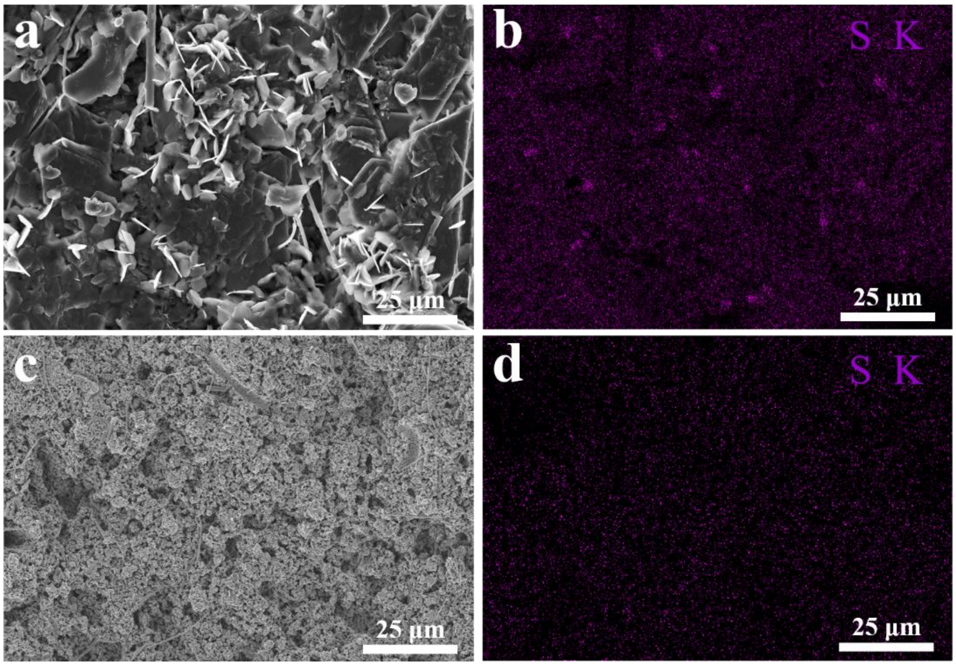

Figure S16. SEM and the corresponding EDS elemental mapping images of (a-b) bare Zn and (c-d) Zn@ZVO electrode after 100 cycles

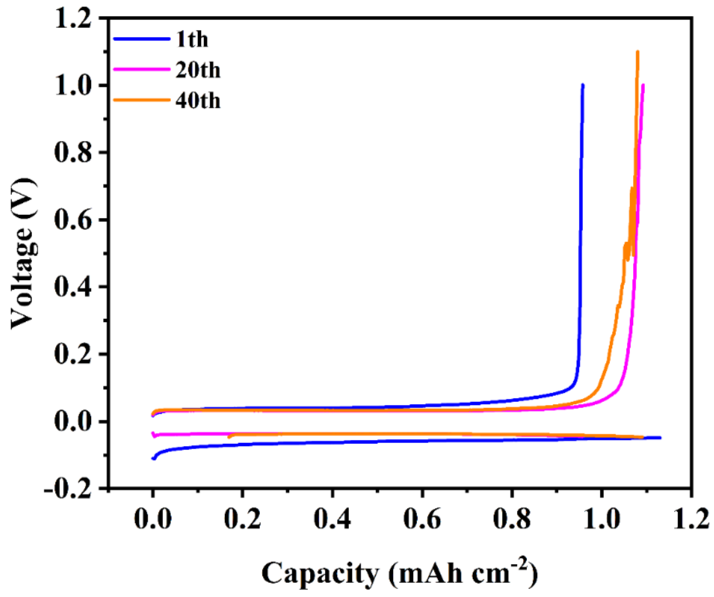

Figure S17. Voltage profiles of bare Ti|Zn cells

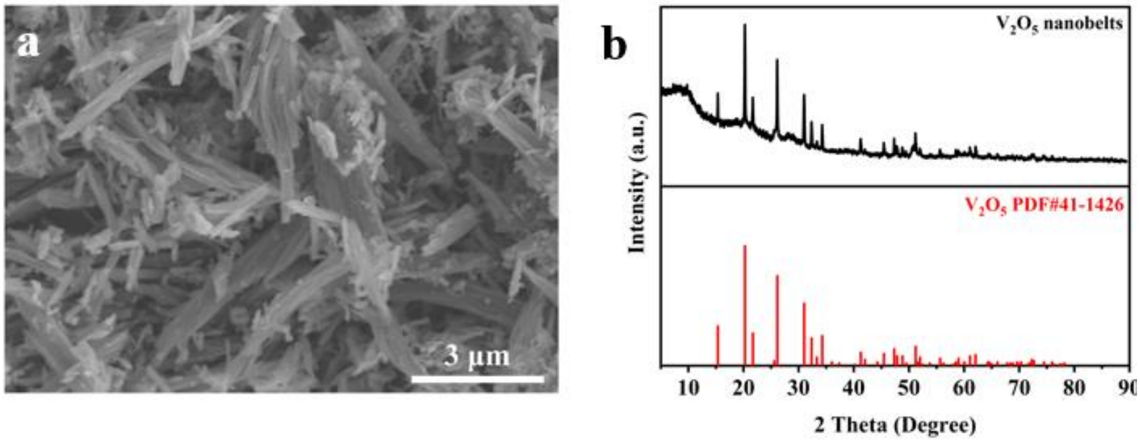

Figure S18. The SEM images (a) and XRD patterns of as-prepared V_2O_5 nanobelts.

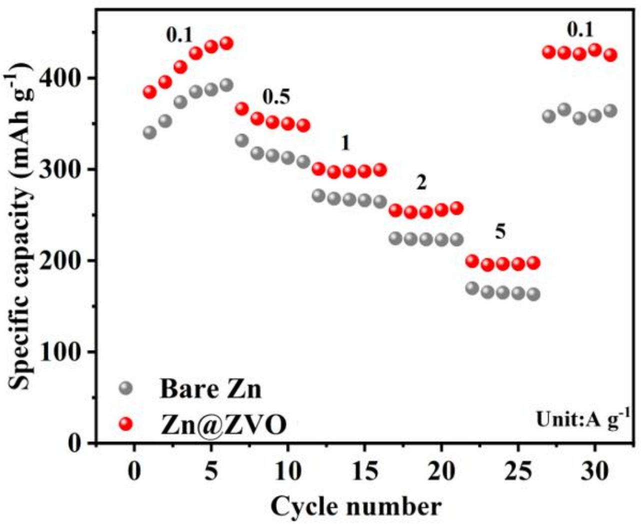

Figure S19. Rate performance of full cells.

Table S1. The charge-transfer resistance (Ω) of the symmetrical cell with bare Zn and Zn@ZVO anode at various temperatures (the equivalent circuit is shown in Figure S8)

Temperatures (°C)	Bare Zn (Ω)	Zn@ZVO (Ω)
25	898.2	253.5
35	504.5	182.8
45	295.1	123.1
55	157.2	80.5
65	81.5	52.0

Table S2. Comparison of electrochemical performance of Zn@ZVO anode with previously reported Zn anodes by solid electrolyte interface coating

Electrode	Current density (mA cm^{-2})	Capacity (mAh cm^{-2})	Life (h)	Cumulative capacity (mAh cm^{-2})	Reference
MMT-Zn	1	0.25	1000	250	[3]
Zn@ZrP	0.5	1	700	700	[4]
rc-SEI@Zn	2	1	750	750	[5]
Zn@LDH	0.5	0.5	1500	750	[6]
Cu-GCN@Zn	0.5	0.25	1000	250	[7]
Zn@Zn-Mont	1	0.5	900	450	[8]
NFZP@Zn	0.5	0.25	2700	675	[9]
NTP@Zn	1	1	240	240	[10]
Nano-CaCO3-coated-Zn	0.25	0.05	800	40	[11]
**Zn@ZVO**	**1**	**0.5**	**2000**	**1000**	**This work**

Table S3. Fitted EIS results of Zn|Zn symmetric cells before cycling

**Electrode**	**R_s (Ω)**	**R_{SEI} (Ω)**	**R_{ct} (Ω)**
Bare Zn	1.29	/	338.4
Zn@ZVO	1.94	18.7	223.5

Table S4. Comparison of electrochemical performance (full cell) of Zn@ZVO anode with previously reported works.

Anode	Cathode	Current density	Cycle number	Initial specific capacity (mAh g^{-1})	Capacity retention	Reference
g-C_3N_4-Zn	CNT/MnO_2	1 A g^{-1}	1300	237.0	61.0%	[12]
Zn@PZA	MnO_2	1 A g^{-1}	700	190.2	95.0%	[13]
rc-SEI@Zn	MnO_2	2 C	690	218.2	78.9%	[5]
EP-Zn	MnO_2	3 A g^{-1}	2000	300.0	50.0%	[14]
	VO_2	2 A g^{-1}	2000	256.0	45.0%	
Zn@ZA	MnO_2	0.2 C	200	240.0	81.2%	[15]
VRM@Zn	MnO_2	1 A g^{-1}	150	160.0	78.4%	[16]
ZnO_x@PC NF/Zn	NMO	1 C	100	93.0	75.0%	[17]
FLG@Zn	MnO_2	10 A g^{-1}	5000	96.5	73.2%	[18]
Zn@Sb	MnO_2	2 C	500	276.0	75.5%	[19]
**Zn@ZVO**	**V_2O_5**	**2 A g^{-1}**	**1000**	**243.8**	**79.1%**	**This work**

References

[1] F. Wan, L. Zhang, X. Dai, X. Wang, Z. Niu, J. Chen, Nat Commun **2018**, 9, 1656.

[2] H. Qin, W. Kuang, D. Huang, X. Zhang, J. Liu, L. Yi, F. Shen, Z. Wei, Y. Huang, J. Xu, H. He, Journal of Materials Chemistry A **2022**.

[3] H. B. Yan, S. M. Li, Y. Nan, S. B. Yang, B. Li, Adv Energy Mater **2021**, 11.

[4] M. Y. Jianping Yan, Yufei Zhang, Yongchao Tang, Cheng Chao Li *, Chemical Engineering Journal **2021**.

[5] H. Yan, C. Han, S. Li, J. Liu, J. Ren, S. Yang, B. Li, Chemical Engineering Journal **2022**, 442.

[6] Y. Yang, C. Liu, Z. Lv, H. Yang, X. Cheng, S. Zhang, M. Ye, Y. Zhang, L. Chen, J. Zhao, C. C. Li, Energy Storage Materials **2021**, 41, 230.

[7] B. Zhou, J. Long, M. He, R. Zheng, D. Du, Y. Yan, L. Ren, T. Zeng, C. Shu, J Colloid Interface Sci **2022**, 613, 136.

[8] L. Hong, X. Wu, C. Ma, W. Huang, Y. Zhou, K.-X. Wang, J.-S. Chen, Journal of Materials Chemistry A **2021**.

[9] S. Wang, Z. Yang, B. Chen, H. Zhou, S. Wan, L. Hu, M. Qiu, L. Qie, Y. Yu, Energy Storage Materials **2022**, 47, 491.

[10] M. Liu, J. Cai, H. Ao, Z. Hou, Y. Zhu, Y. Qian, Adv Funct Mater **2020**, 30, 8.

[11] L. Kang, M. Cui, F. Jiang, Y. Gao, H. Luo, J. Liu, W. Liang, C. Zhi, Adv Energy Mater **2018**, 8, 1801090

[12] X. Wang, K. Yang, C. Ma, W. Lu, N. Chen, M. Yao, Z. Li, C. Liu, H. Yue, D. Zhang, F. Du, Chemical Engineering Journal **2023**, 452, 139257.

[13] J. Wang, Z. Zhao, F. Hu, H. Song, Q. Xie, X. Wan, S. Song, Chemical Engineering Journal **2023**, 451, 139058.

[14] R. Zhu, Z. Xiong, H. Yang, T. Huang, S. Jeong, D. Kowalski, S. Kitano, Y. Aoki, H. Habazaki, C. Zhu, Energy Storage Materials **2022**.

[15] L.-F. Zhou, X.-W. Gao, T. Du, H. Gong, L.-Y. Liu, W.-B. Luo, Journal of Alloys and Compounds **2022**, 896.

[16] B. Zhou, A. Hu, X. Zeng, M. He, R. Li, C. Zhao, Z. Yan, Y. Pan, J. Chen, Y. Fan, M. Liu, J. Long, Chemical Engineering Journal **2022**, 450.

[17] Y. Song, Y. Chen, Z. Wang, W. Zhao, C. Qin, H. Yu, X. Wang, Z. Bakenov, Y. Zhang, Journal of Power Sources **2022**, 518.

[18] M. Qiu, H. Jia, H. Liu, B. Tawiah, S. Fu, Journal of Alloys and Compounds **2022**, 891.

[19] L. Hong, L. Y. Wang, Y. Wang, X. Wu, W. Huang, Y. Zhou, K. X. Wang, J. S. Chen, Adv Sci (Weinh) **2022**, 9, e2104866.
